# A decision aid to support family carers of people living with dementia towards the end‐of‐life: Coproduction process, outcome and reflections

**DOI:** 10.1111/hex.13307

**Published:** 2021-07-19

**Authors:** Nathan Davies, Elizabeth L. Sampson, Emily West, Tanisha DeSouza, Jill Manthorpe, Kirsten Moore, Kate Walters, Karen Harrison Dening, Jane Ward, Greta Rait

**Affiliations:** ^1^ Department of Primary Care and Population Health, Royal Free Campus University College London London UK; ^2^ Marie Curie Palliative Care Research Department University College London London UK; ^3^ Barnet, Enfield and Haringey Mental Health Trust, Liaison Psychiatry Team North Middlesex University Hospital London UK; ^4^ NIHR Policy Research Unit in Health and Social Care Workforce, Policy Institute at King's King's College London London UK; ^5^ NIHR Applied Research Collaborative (ARC) South London King's College London London UK; ^6^ National Ageing Research Institute Parkville Victoria Australia; ^7^ Dementia UK & Admiral Nursing London UK; ^8^ Family Carer, Member of Experts by Experience Panel

**Keywords:** carers, codesign, coproduction, decision‐making, dementia, end‐of‐life care

## Abstract

**Background:**

Family carers of people living with dementia often need support with making decisions about care. Many find end‐of‐life care decisions particularly difficult. The aim of this article is to present an evidence‐ and theoretical‐based process for developing a decision aid to support family carers of people with dementia towards the end‐of‐life.

**Methods:**

Following a systematic process, we developed a decision aid using coproduction methods and matrices to synthesize data from a systematic review and qualitative interviews with people living with dementia and family carers. Data were presented to coproduction workshops of people living with dementia, family carers, practitioners and professionals. Development was guided by the Ottawa Decision Support Framework and a modified Interprofessional Shared Decision‐Making model.

**Results:**

The decision aid covers four decision areas: (1) changes in care; (2) eating and drinking difficulties; (3) everyday well‐being; and (4) healthcare, tests and medication. We present an interactive decision aid, using a variety of approaches including written text, Frequently Asked Questions, top tips and illustrative quotes from people living with dementia and family carers.

**Conclusion:**

This is the first decision aid that focusses on multiple decisions towards the end‐of‐life in dementia care. The process offers a template for others to develop decision aids or similar interventions, and how to include people living with dementia in coproduction.

**Patient or Public Contribution:**

Family carers provided feedback on data collection, data analysis and the decision aid, and one is a coauthor. People living with dementia and family carers were integral to the coproduction workshops.

## 1 BACKGROUND

Most people living with dementia live in the community, in their own homes or with family.^1^, ^2^ Approximately 700,000 family members and friends are primary carers providing the majority of care for people living with dementia in the United Kingdom.^3^ For the purpose of this study, we used the term family carer to describe family and friends providing unpaid care for someone living with dementia, acknowledging that they may not identify themselves as a carer.^4^, ^5^

Shared decision‐making is an important part of healthcare and person‐centred dementia care.^6^ However, as an individual's dementia progresses, he or she becomes less likely to be able to make decisions about his or her care, well‐being and general welfare. Advance care planning (ACP) is a process of planning for care in the future. Studies have demonstrated the effectiveness of ACP on several outcomes including family satisfaction with care and burdensome transitions in care.^7^, ^8^ However, many individuals reach the point when they no longer have capacity to make their own decisions. In England and Wales, under the Mental Capacity Act (MCA) 2005, decisions for those who lack capacity should be made in their best interests if there is no prior legal authority.^9^ Decision‐makers will vary and are usually made through a family carer or a health or care professional depending on the significance of the decision to be made and national legislation; however, a shared decision‐making approach should be encouraged.

Family carers find decisions or discussions about severe dementia and end‐of‐life difficult, such as stopping treatment,^10^, ^11^, ^12^ and they may benefit from support in making decisions.^13^, ^14^, ^15^ Help may be welcomed by carers making significant decisions, especially about support when their family member is in the severe stages of dementia and towards the end‐of‐life.^16^, ^17^ A recent ACP intervention in nursing homes has been shown to be effective in reducing family carer uncertainty with decision‐making, and improving perceptions of quality of care.^18^ However, this approach includes a facilitator and is based in nursing homes, where there may be more support to complete ACP than for carers at home with the person living with dementia. It has been recognized that alternative approaches to decision‐making are required across different settings.^7^

Another approach to support family carers is decision aids, which guide the decision‐maker through different stages of a decision. These can take various forms including booklets, pamphlets, videos or web‐based tools. Decision aids provide information about the decision and summarize options along with associated benefits and harms to enable people to make and document decisions.^19^ There is substantial evidence from the health sector demonstrating the effectiveness and feasibility of decision aids to support decision‐making.^19^, ^20^

The clarity about the options that decision aids offer may be particularly useful for family carers of people living with severe dementia or those approaching the end‐of‐life. Such times often involve a variety of symptoms and comorbidities, potentially creating confusion and a multitude of difficult decisions for the family carer.^21^

Decision aids suitable for use by family carers in dementia care have been shown to improve knowledge and communication, and reduce decisional conflict among family carers.^16^, ^22^ However, these decision aids only focus on a single decision, including eating and feeding options,^23^ place of care^24^, ^25^ and goals of care.^26^, ^27^ When caring for someone living with dementia towards the end‐of‐life, family carers are often faced with multiple decisions, and these are often inter‐related. There is currently no decision aid for dementia care that covers multiple decisions. There is a need for holistic decision aids to reflect this complexity.^16^ In particular, studies have identified a need for decision aids that focus on those with dementia towards the end‐of‐life.^16^, ^17^

It is important that end users (family carers), together with those who the decision aid affects (people living with dementia) and those who will interact with carers using the decision aid (practitioners and carer organizations/advocates), are involved in development. However, reviews of patient decision aids have highlighted a lack of clear reporting about the development process of decision aids.^16^, ^28^

This article reports the systematic development and components of a decision aid for family carers of a person living with dementia towards the end‐of‐life. This builds on a programme of work that included a systematic review of evidence for effectiveness of existing decision aids,^16^ and a qualitative study with family carers of people with dementia towards the end‐of‐life and people living with dementia themselves.^29^

The aim of this article is to present an evidence‐ and theoretical‐based process for developing a decision aid to support family carers of people with dementia towards the end‐of‐life.

## 2 METHODS

### 2.1 Design

A coproduction approach was adopted to develop a decision aid, as part of a systematic development process for decision aids.^28^, ^30^

Coproduction is increasingly being used in healthcare research and for the development of resources used in clinical practice, including in dementia research.^31^, ^32^, ^33^ Partnership with stakeholders and end users has been identified as a fundamental approach of intervention development and this is the core premise of coproduction.^34^ The use of coproduction is particularly recommended for use in the development of patient decision aids.^35^

We were informed by a review of developing complex interventions,^34^ which provides a comprehensive range of approaches and actions to develop complex interventions. Actions are described across the domains of conception, planning, designing, creating, refining, documenting and planning for future evaluation.

### 2.2 Theoretical frameworks underpinning development

The development of content for the decision aid was guided by two theories: (1) the Ottawa Decision Support Framework (ODSF)^36^ and (2) a modified version of the InterProfessional–Shared Decision Making (IP‐SDM) Model.^37^

#### 2.2.1 ODSF

The ODSF was developed to help facilitate shared decision‐making between patients and healthcare professionals.^24^, ^36^ Decision aids developed using the ODSF have been shown to be effective in reducing decisional conflict and improving decisional quality.^20^ We chose this framework as it supports health decisions that lack clear choice, are value laden, require much deliberation and are subject to changing circumstances.^36^ This resonates with the decisions that carers of people with dementia encounter. The aim of the ODSF is to improve the quality of decision‐making by addressing modifiable and suboptimal determinants of decisions, including unrealistic expectations, unclear views, unclear norms, unwanted pressure, inadequate support and inadequate personal and external resources to make the decision. ODSF is organized into (1) decisional needs, (2) decision support and (3) decision quality (see Table 5). It states that the decisional needs will affect the quality of the decision, and this can be mediated by decision support such as a decision aid.

#### 2.2.2 The IP‐SDM model

The IP‐SDM model has been widely used to inform development of decision aids;^38^ it conceptualizes the decision‐making process and the factors that influence this.^37^ We used a modified model based on the dementia population, who lack capacity, and the specific decisions facing family carers.^29^ The model consists of two sections: the context in which decisions are made and the decision‐making process. The context considers personal preferences, ACP and legal aspects of decision‐making, health and well‐being of the individual (including capacity), support from others and clarity of decision‐maker roles. The process consists of (1) identifying the decision‐maker or team, (2) sharing and exchanging information, (3) clarifying values and preferences, (4) managing and considering emotions, (5) considering the feasibility of options, (6) balancing the preferred choice and the actual choice, and (7) implementation and reflecting on outcomes.

#### 2.2.3 International patient decision aid standards

We report the content and development of the decision aid against the International Patient Decision Aid collaboration set of Standards and criteria (IPDASi v 4.0) for decision aids^39^ (see appendix).

### 2.3 Ethics statement

This study received ethical approval from the London Queen Square Research Ethics Committee (18/LO/0408), and all participants provided written informed consent.

### 2.4 Participants and recruitment

#### 2.4.1 Family carers and people living with dementia

Eight family carers of people living with dementia who identified as caring for someone towards the end‐of‐life, and four people with mild dementia were purposively sampled.

Family carers and people with dementia were recruited via clinical services including National Health Service (NHS) memory clinics and general practices in Greater London, UK. Invitations were sent by the recruiting organization, and clinical teams reviewed the eligibility criteria before inviting participants. Interested participants were asked to contact the research team directly. Recruitment was supplemented from local and national dementia organizations, National Institute for Health Research (NIHR) Join Dementia Research (online network) and participants from earlier phases of the project.

#### 2.4.2 Practitioners

Eleven practitioners who provided care for people with dementia towards the end‐of‐life and from carer support organizations were recruited through contacts of the research team and supplemented with snowballing methods. Participants were purposively sampled for a variety of occupations.

### 2.5 Inclusion and exclusion criteria

All participants had to be able to read and speak English and provide informed written consent.

#### 2.5.1 Family Carers

*Inclusion criteria*

- Family member or friend who provided unpaid care for a person living with dementia in the later stages of dementia or towards the end‐of‐life.
- Current or former carer.
- Proxy decision‐maker for the person living with dementia, either informally or through lasting power of attorney for health and welfare.
- Over the age of 18 years.
*Exclusion criteria*
- Carers bereaved in the past 3 months.

#### 2.5.2 People with dementia

*Inclusion criteria*

- Clinical diagnosis of any type of dementia.
- Mental capacity to provide informed consent.
- Over the age of 65 years.

#### 2.5.3 Practitioners from health and care services and carer organisations

*Inclusion criteria*

One or more of the following:

• Practitioners in a caring role (health or social care), for someone with dementia.
• Experienced in providing end‐of‐life care and contributing to decision‐making dementia or family carers.
• Experienced in working directly with people living with dementia or family carers.

### 2.6 Procedure

**Figure 1:**
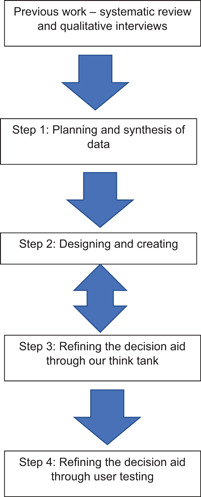
Overview of the development process

An experts by experience group consisting of four family carers oversaw the whole project, commenting on study documents, procedures and findings. An overview of the process and procedure is provided in Figure 1.

#### 2.6.1 Earlier phases of the overall project

The systematic review^16^ and qualitative data collection^29^ in earlier parts of the project allowed us to complete the following actions from O'Cathain et al.'s taxonomy^34^:

- understand the problems or issues (decisions) to be addressed;
- understand the experiences of family carers and people living with dementia about decision‐making;
- understand the perspectives and the psycho‐social context of family carers making decisions;
- understand the wider context of decision‐making in dementia care;
- consider where the decision aid will be implemented; and
- identify evidence of the effectiveness of similar interventions.

The qualitative data highlighted the key decisions to be made and the factors to consider in the decision‐making process. Key decisions included (1) transitions in care; (2) medical care or clinical interventions, and physical well‐being; (3) eating and drinking; (4) psychological/emotional well‐being; (5) distress of the individual (including emotional well‐being); (6) communication; and (7) conflict/disagreement with others about providing care and decisions. There were multiple factors that needed to be considered when making decisions including personal preferences, the emotional experience of decision‐making, the health and well‐being of the person living with dementia, professional input and support. Finally, these data were used to revise the IP‐SDM model and provide the seven steps of the decision‐making process detailed in the IP‐SDM section above.^29^

The systematic review helped to identify existing decision aids to share with the coproduction groups, understand what components were included in previous decision aids and which decision aids were effective.^16^ The review concluded that decision aids developed thus far have focussed on single decisions. Further decision aids are required that reflect the complexity of dementia care with multiple decisions to be made by family carers, which are often inter‐related.

Coproduction was divided into four main steps:

Step 1: Planning and synthesis of data.

To synthesize the findings from the qualitative data and systematic review, we constructed a series of matrices to allow a transparent and thorough mapping of the different sources of data. This process was iterative and on‐going, before and during the coproduction workshops, and when refining the decision aid. After each workshop, key points were summarized in Table 1. This provided a clear overview of the potential topics and decisions to be included in the decision aid.

**Table 1:** Matrix completed iteratively before and during the Coproduction process

Subdecision (Table 1)	Factors influencing decision‐ making	Format to address decision	Source of evidence (systematic review, qualitative study or published evidence)	Coproduction workshop recommendation	Include (yes/no)
Example 1: Difficulties with person refusing medication	Benefit of medication Person's wishes	Examples of experiences from other people	Qualitative study	Factors related to the benefits of the medication need to be considered and what the impact would be without the medication.	Yes

Step 2: Designing and creating.

We constructed a series of coproduction groups to meet in workshops.

#### 2.6.2 Aim of workshops

Based on O'Cathain et al.'s taxonomy,^34^ the aims of the workshops were to understand wider stakeholders' perspectives of the decisions and issues, decide upon the specific decisions that the decision aid should address, and the aims or goals for the decision aid; consider real‐world delivery of the decision aid; decide on the content, format and delivery; and make prototypes of the decision aid.

#### 2.6.3 Composition of the workshops

We created four homogeneous coproduction groups: people living with mild dementia, family carers and two groups of professionals from health and social care services (see Tables 2, 3–4).

**Table 2:** Practitioners from health and care services and carer organizations

Role	*N*
End‐of‐life care facilitator	1
Memory service nurse	1
Occupational therapist	1
Psychologist	2
Nurse practitioner	1
Social care professional	1
Palliative care nurse	1
Health editor	1
Clinical educator–palliative care	1
Dementia support worker	1
Gender	
Male	2
Female	9

**Table 3:** Family carer demographics

	*N*
Relationship to person with dementia	
Spouse	3
Sibling	1
Adult child	4
Gender	
Male	2
Female	6
Current or former	
Current carer	7
Former carer	1
Ethnicity	
White British	6
Mixed	1
Pakistani	1

**Table 4:** People with dementia demographics

	*N*
Type of dementia	
Posterior cortical atrophy	1
Not known	2
Frontotemporal	1
Gender	
Male	3
Female	1
Age	
60–69	1
70–79	1
80–89	1
Unknown	1
Ethnicity	
White British	3
Taiwanese	1

#### 2.6.4 Including people living with dementia in coproduction workshops

We worked closely with our experts by experience group and practitioners working in dementia care to carefully consider how to optimally run a workshop with people living with dementia. We ensured that this workshop consisted of a small number of participants (e.g., 3–5), to minimize distractions and confusion. We wanted to ensure a feeling of being in a safe environment and of feeling comfortable throughout, developing trust and a rapport with researchers.^40^ To establish this trust and safe environment, we offered the option of bringing a person with them such as a family carer. However, to ensure that the contributions of people living with dementia were heard, we asked, if possible, that the other person might sit in an adjacent room. As most participants had taken part in the qualitative phase of the study, at least one of the researchers was familiar to them. We also held a preworkshop telephone discussion to provide information about the study and build on the rapport and relationship with the participant. At the start of the workshop, we spent time talking and ‘mingling' over refreshments to create a calm and relaxed atmosphere.

We provided accessible (large print and plain English) printed worksheets and information to support participants living with dementia. We ensured that a third researcher was available to sit with the carers who attended. Participants were also given the option to use a printed coloured card that read ‘I wish to speak' to hold up if they were not able to speak out or felt that they could not be heard. Finally, we provided breaks during the workshop and ensured that the timing was flexible to meet individuals' needs, following others' recommendations.^31^, ^41^

#### 2.6.5 Workshops' structure and contents

Each group met once for 1.5 h over several months as workshops facilitated by two or three researchers (N. D., T. D. S.). We used a modified nominal group process,^32^, ^42^, ^43^ which includes using structured meetings to solve specific problems, facilitate group thinking and decision‐making. Participants were presented with short inputs on background, reviews of literature and evidence from the qualitative interviews through PowerPoint presentations that were based on the matrices. We used a combination of approaches and stages to encourage discussion and generate ideas (see Box 1). Detailed notes were made throughout the discussion, and participants provided additional feedback via email. At the end of each workshop, the research team agreed with the group on the key points to inform the next iteration of the decision aid.

Box 1. **Stages of workshops**

Stage 1: Welcome and overview of project, overview for workshop, aims of this group and ground rules.

Stage 2: Presentation and discussion of example decision aids (Carers workshop only).

Stage 3: Key findings from the qualitative study (presented as 10 key decisions and factors influencing decisions).

Stage 4: Discussion of each decision facilitated by key questions.

Stage 5: Rank priority of decisions.

Stage 6: Presentation and discussion about developing the prototype.

Stage 7: Summary of discussion and close.

We used the IP‐SDM model to shape our discussions in the workshops on the format of the decision aid. For example, we asked participants how to present in the decision aid an opportunity to reflect on the wishes, preferences and values of the person with dementia when making decisions.

In the first workshop with carers, we provided and discussed examples of decision aids identified from our systematic review. In the other three workshops with the two professional groups and with people living with dementia, we showed participants the developing prototype of our decision aid. This group order ensured that the core topics and contents of the decision aid were grounded in the views of family carers and people living with dementia, placing greater emphasis on these groups as the ultimate end users. Professional groups helped us to flesh out the content including clinical details and advice to add to the decision aid.

Step 3: Refining the decision aid through our think tank.

In parallel to Step 2, Step 3 focussed on refining and finalizing the decision aid. A fifth group acting as a ‘think tank' to support coproduction was created. This group consisted of two family carers, a General Practitioner (GP), an Admiral Nurse (specialist dementia nurse that support families), two gerontologists and a social work expert. The research team also joined this group, consisting of two psychologists, a GP and an Old Age Psychiatrist. The detailed notes were summarized and presented to the group. In particular, the group:

- provided clarity about ideas and discussions from coproduction workshops;
- ensured that the research team considered the views from all three coproduction groups; and
- ensured that the decision aid was grounded in evidence.

The fifth group met three times for 1–2 h, in between the workshops.

Step 4: Refining and user testing.

Once the decision aid was finalized by the ‘think tank,' it was tested with the experts by the experience group and two carers from the coproduction workshop. They read through the decision aid with one individual researcher, and using the principles of the think aloud method,^44^ provided their initial impressions of the format and content of the decision aid.

The results from the user testing were discussed among the core research team (N.D., G.R., E.L.S.), and comments were incorporated.

## 3 FINDINGS

### 3.1 Overview of the decision aid

**Figure 2:**
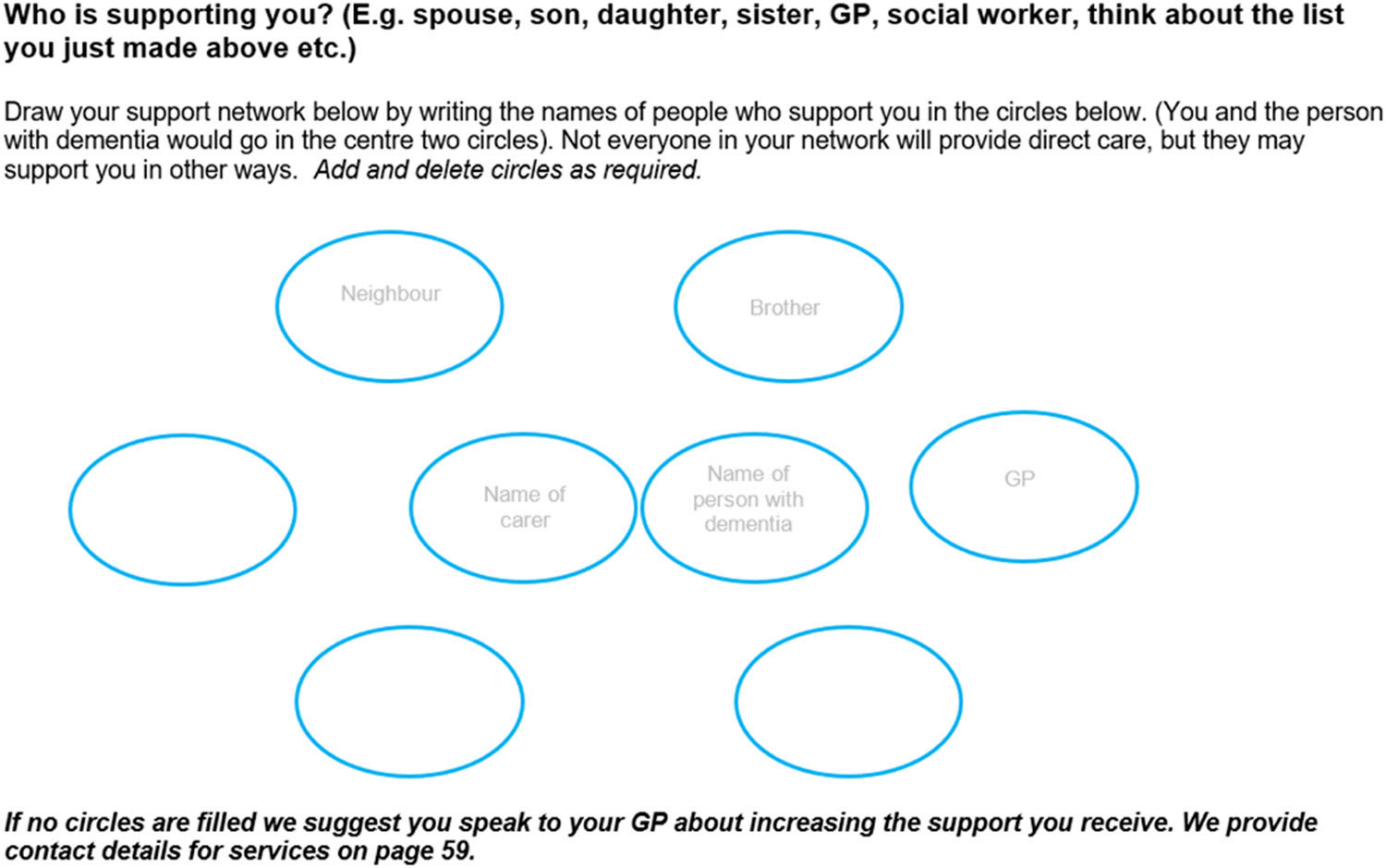
Identifying carers' support networks

The decision aid opens with an introductory section about dementia, end‐of‐life care and considering the context of the decision‐maker, including clarifying what their role is, and what is important to them. Subsequently, carers record their social network, including who they see on a daily basis, and record this on a social network diagram (see Figure 2).

Participants at all workshops considered that it was important to highlight who was responsible for making decisions, and to emphasize that decisions were not necessarily the family carers' to make, in particular, decisions about medical care and treatment. To highlight this, the decision aid includes information on ‘best interests' decisions, asks the carer if they have Lasting Power of Attorney and encourages discussions with health and social care teams throughout. A further section discusses the importance of looking after themselves as carers, and advice about staying well.

### 3.2 Decisions

The workshops were presented with seven decisions from the qualitative data. After the initial discussion, workshops ranked the decisions based on importance and relevance to include in the decision aid. This helped to consider decisions in turn to inform subsequent discussions. Carers in the workshops were not surprised by any of the decisions that we presented.

The decision regarding conflict/disagreement with others, followed by communication, and eating and drinking received the lowest scores across all workshops. Discussion within the workshops revealed that communication and conflict were more contextual factors that influence all decisions. However, managing eating and drinking were considered a decision that was important to include despite its low ranking, across all groups. Conflict and communication were removed as decision topics, but were added as background information sections.

During a meeting with the ‘think tank', researchers fed back on the discussions and ranking from the workshops. Four decisions were chosen as the final decisions to include in the decision aid:

1. Changes in care.
  The qualitative data highlighted the challenges to family carers of transitions in care, including moving into a care home, hospital admissions and changes in the level of home care. The systematic review had found decision aids developed for decisions about care home moves, but little on other aspects of care transitions and home care. Evidence from the interviews and systematic review was presented to the coproduction groups. All agreed that this was one of the most important and common decisions. Carer and professional coproduction groups highlighted the need to include information for both carers who were self‐funded and state funded. Carers were also keen to highlight the need to consider what they as carers could manage and recognize their preferences.
2. Eating and drinking difficulties.
  Decision aids covering eating and drinking were identified in the systematic review; however, they focussed on artificial nutrition and hydration. Qualitative data from family carer interviews showed that they felt ill equipped and under‐informed more broadly about eating and drinking. This included understanding the progression of dementia and its impact on eating and drinking, and the importance of a healthy diet. In the coproduction workshops, professionals viewed this as more of a challenging area than carers; however, many carers may not have encountered such problems. The decision aid provides ‘myth busters' about eating and drinking, including tips on how to manage this, such as how to encourage eating. Carefully worded information is provided to explain that people eat less towards the end‐of‐life and it is not important to encourage a healthy diet, but rather food they find enjoyable.
3. Everyday well‐being for the person with dementia.
  Ensuring the health and well‐being of the individual was a prominent theme in the qualitative data. This encompassed not only increasing physical needs as the individual's dementia progressed but also their emotional well‐being. Originally two separate decisions, the carer coproduction group felt that physical well‐being and psychological/emotional well‐being could be encapsulated as everyday well‐being for the person living with dementia in the decision aid. Carers from workshops were keen to highlight continence as a specific challenge. Professionals emphasized that continence was often the key ‘tipping point' for many carers to stop continuing to provide care at home. Other aspects included sleep and distress.
4. Healthcare, tests and medication.

The systematic review identified decision aids focussed on goals of care, and qualitative data described the various tests and treatments that participants did not want or felt needed to be carefully considered if they were approaching death. The coproduction groups with people living with dementia and professionals were keen for the section to highlight that many of these decisions are not just the carers' responsibility, but should be a shared decision‐making approach with the healthcare team. A summary table of medications is provided for carers to complete with the healthcare team to encourage discussion and de‐prescribing as necessary (see Appendix).

### 3.3 Format and engagement

Directed by the seven stages of the adapted IP‐SDM model, we broke down the decision‐making process for each of the four decisions. Throughout these seven stages, we carefully followed the ODSF to inform the content. Tables 5 and 6 provide an overview of how we operationalized the ODSF and IP‐SDM models by using a variety of components in the decision aid.

**Table 5:** Formatting of the decision aid to ensure that we met the ODSF steps and how we operationalized them in practice

	Clarify decision and needs	Provide facts, probabilities	Clarify values	Guide in deliberation and communication	Monitor/facilitate progress
Introductory information	**X**	**X**		**X**	
List of options		**X**			
Benefits and disadvantages		**X**	**X**		
Myth busters		**X**			
Top tips		**X**		**X**	
FAQ		**X**			
Questions for GP	**X**			**X**	
Charting medication				**X**	
Questions to respond to	**X**		**X**		**X**
Stories about others' experiences				**X**	
Quotes from others				**X**	
Detailing support network				**X**	

Abbreviations: FAQ, frequently asked question; GP, General Practitioner; ODSF, Ottawa Decision Support Framework.

**Table 6:** Formatting of the decision aid to ensure that we met the steps in the IP‐SDM model, and how we operationalized these steps in practice

	Identifying the decision‐maker or team	Sharing and exchanging information	Clarifying values and preferences	Managing and considering emotions	Considering the feasibility of options	Balancing the preferred choice and the actual choice	Implementation and reflecting on outcomes
Introductory information	**X**	**X**		**X**	**X**		
List of options		**X**			**X**	**X**	
Benefits and disadvantages		**X**	**X**	**X**	**X**	**X**	
Myth busters		**X**		**X**	**X**	**X**	
Top tips		**X**		**X**			
FAQ		**X**					
Questions for GP		**X**			**X**	**X**	**X**
Charting		**X**					
Questions to respond to			**X**	**X**	**X**	**X**	**X**
Stories about others' experiences	**X**	**X**		**X**	**X**		
Quotes from others	**X**	**X**		**X**	**X**		
Detailing support network	**X**			**X**			

Abbreviations: FAQ, frequently asked question; GP, General Practitioner; IP‐SDM, InterProfessional–Shared Decision Making.

**Figure 3:**
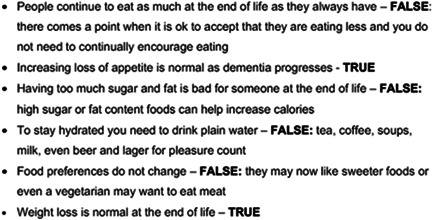
Myth busters examples from the eating and drinking decision section

Engagement with both the person living with dementia and family carer was a key consideration throughout our coproduction and supported by our adapted IP‐SDM model. The carers group thought that it was vital that there was space in each of the decisions to reflect on the consequences and outcomes of each decision. The resulting decision aid is presented as an interactive booklet to engage carers. To encourage engagement, the decision aid poses questions to carers throughout. Carers record their answers and preferences. Information is provided via a variety of formats including written text, frequently asked questions (FAQs), top tips and illustrative quotes from people living with dementia and family carers. We provide myth busters as recommended by professionals and supported by family carers and people with dementia in our coproduction groups (see Figure 3). These formats were seen as a way to engage the reader while demonstrating the key principles of a decision aid as defined in the IPDASi v4.0 including describing the options available, the benefits and advantages of options and experiences of the consequences of the options (e.g., physical, psychosocial and social).

Based on family carer suggestions, we included individual stories or broad scenarios from people who have experienced similar situations, with information about how they came to their decision and the outcomes. Participants felt that carers would relate more to a story than just facts. This approach was used for discussing continence, an often embarrassing topic.

Following suggestions from the professionals workshop, we included the benefits and disadvantages of options using an activity asking carers to list all the reasons why they would choose the option (i.e., benefits) and reasons why not to choose this option (i.e., disadvantages) on a set of blank paper cards provided with the decision aid. These cards can then be stacked into two piles to help visualize which options/preferences have the most benefits and disadvantages.

Finally, carers are asked if they have enough information and, if yes, which preference or option they prefer, recording their decision. If they do not have enough information to make a decision, the final section for each decision provides both (1) signposting to specific groups and organizations and (2) a space to write questions to discuss with a health or care provider. Throughout the decision aid, carers are encouraged to discuss options and decisions with other members of their support network, including health and social care professionals, ensuring a shared decision‐making approach.

## 4 DISCUSSION

This article presents a novel decision aid to support family carers of people living with dementia towards the end‐of‐life. This is the first decision aid to cover multiple decisions that family carers may face when a person living with dementia is approaching the end‐of‐life, filling an important gap identified in the decision‐making literature.^7^, ^16^, ^22^ This is one of the first papers to provide a detailed description of a systematic approach to coproducing a decision aid, grounded in theory, evidence and lived experience. There is increasing recognition of the importance of high‐quality reporting of coproduction methods and the development process of decision aids.^45^, ^46^, ^47^ Few decision aids developed in dementia care report their development clearly.^16^ We have provided clarity on how data from multiple sources including theory can be synthesized and presented to end users and meaningfully contribute to coproduction. This transparent method will be helpful to researchers developing complex interventions through coproduction. Importantly, this study is one of the first to include people living with dementia in coproduction, and we provide key learning points.

Family carers have reported feeling ill‐prepared for decisions about end‐of‐life care and a lack of professional support.^48^ The course of dementia is unpredictable, and goals of care and preferences may change over time^49^; therefore, it is important to acknowledge the complexities of decision‐making and the need for carers to be adequately supported. Decisions will need to be revisited and may change over time^50^; our decision aid encourages carers to revisit decisions and to discuss with professionals and other family members, which can enhance decision‐making.^51^ Importantly, the decision aid highlights that decisions are not just the carer's responsibility, but should be part of a shared decision‐making approach with the care team.^52^

The decision aid is an interactive booklet using multiple approaches to engage participants and encourage reflection, moving beyond simple provision of information. The decision aid focusses on support across changes in care, eating and drinking difficulties, everyday well‐being in dementia and healthcare tests and medication. It was important that we provided information and options across these decisions in a balanced and neutral manner,^53^ providing evidence‐based information.

One of the unique features of the development of this decision aid was the inclusion of people living with dementia in the design of the intervention. In the methods, we have highlighted our approach to ensuring that they were adequately supported to participate. Previous work has developed alternative methods to increase the inclusivity and engagement of people in coproduction,^46^ but considering this in the context of living with dementia is important. There are increasing numbers of articles reporting on the inclusion of people with dementia in research; however, they lack detail on coproduction methods.^54^ Importantly, we consider that some of the key lessons from our experience include ensuring people living with dementia feel comfortable, acknowledged in the research procedures and have the time to contribute meaningfully. These can be achieved through minor modifications to research procedures including holding smaller groups, reducing the number of tasks in workshops, increasing the number of researchers to provide support (this may need to be one to one), considering the cognitive load of tasks and using accessible documents including consent and information sheets.

We prepared tasks for participants to rank the priority of decisions to be included. This was well received by family carers, although many felt that some were equally important. Professionals felt that many of the topics were not isolated decisions and they were difficult to rank. This reflects the clinical reality of having to consider multiple challenges rather than a clear ranked list of priorities. People living with dementia found this task demanding. We provided one‐on‐one support for this task, but they continued to struggle, and on reflection, this was a cognitively demanding task that we would not recommend for future workshops with people living with dementia. Tasks that involved visual representations proved much easier, including providing participants with an example of our prototype decision aid.

### 4.1 Implications for research

Research needs to further explore optimal methods and approaches for including people living with dementia in decision‐making research and end‐of‐life research.

Our article provides an example of how a matrix approach can be used to synthesize findings and for intervention development. This is a vital step that is missing in guidance on developing complex interventions.^28^, ^55^ It is an iterative approach that provides transparency and clarity.

The decision aid developed will be tested in a feasibility study exploring optimal methods of evaluation and considering acceptability. We will continue to work with end users and stakeholders in the evaluation, which is vital to support implementation in routine care and practice.^56^ Finally, it is also important to consider in future research the inclusion of those who do not speak English; this will allow us to tailor the decision aid to these populations.

### 4.2 Implications for policy and clinical practice

This decision aid has many potential benefits for family carers, people living with dementia and health and social care practitioners. The aid could be distributed by GPs in consultations with family carers, for example, supporting shared decision‐making, which is a priority for the NHS Long Term Plan.^57^ For carers, the decision aid may help to clarify decisions, the options available and the decision‐making process, reducing decisional conflict, feelings of guilt and potentially help prepare them for their relative's end‐of‐life. Despite the decision aid being developed for family carers towards end‐of‐life, it may be beneficial for people living with dementia to use for ACP.

### 4.3 Strengths and limitations

We used a systematic approach following the principles of an evidence‐based process for developing decision aids.^28^ To supplement the gaps in guidance about specific approaches to design of complex interventions, we used a taxonomy of evidence‐based approaches from O'Cathain and colleagues.^34^

Our decision aid is strengthened by the theoretical foundations, using the IP‐SDM model modified for dementia care decisions and the ODSF.^24^, ^36^ These enabled us to break down complex decisions and provide a clear pathway for carers using the decision aid. Furthermore, this was supplemented by experts from across clinical and academic fields, but most importantly, included people living with dementia and family carers in the design process.

The matrix‐based approach for synthesis offered a transparent and systematic approach to synthesis, ensuring that the development was rigorous and evidence based.

Due to the size of coproduction workshops, we were unable to include all professional roles that may have been relevant. For example, the inclusion of pharmacists, speech and language therapists and dieticians would have been beneficial and should be targeted in future research. Furthermore, we were not able to include people who did not speak English; therefore, we may have missed some important cultural variations and factors that need to be considered when making decisions with those who do not speak English.

## 5 CONCLUSION

This article presents the first decision aid that focusses on multiple decisions towards the end‐of‐life for people living with dementia. We provide a detailed overview of the systematic development process of a decision aid for family carers of people living with dementia. The process offers a template for others to develop decision aids or similar decision‐making interventions, and how to include people living with dementia in coproduction.

## CONFLICT OF INTERESTS

The authors declare that there are no conflict of interests.

## AUTHOR CONTRIBUTIONS

Nathan Davies conceived the idea and design of the study and acquired funding, acquired data, led analysis and interpretation of the results and drafted and finalized the manuscript. Elizabeth L. Sampson and Greta Rait conceived the idea and design of the study and acquired funding, and assisted in data analysis and interpretation. Emily West, Jill Manthorpe, Kate Walters, Kirsten Moore, Karen Harrison Dening and Jane Ward contributed to data analysis and interpretation. Tanisha DeSouza acquired data, and contributed to analysis and interpretation. All authors contributed to drafting the manuscript and revising for critically important intellectual content. All authors have approved the version to be published and agree to be accountable for all aspects of the work.

## Data Availability

Data sharing not applicable to this article as no data sets were generated or analysed during the current study.

## APPENDIX A Medication charting

Make a list of their medications. Discuss the list with their GP or pharmacist. Consider do they still need this medication? Some medications they are taking may no longer be needed or helpful.

You can use the questions in this table as a basis for your discussion and complete together.

**Table:**

Medication	Purpose	How often do they take it?	When was it prescribed or reviewed? (should be at least the last 12 months)	How you noticed any side effects?	Continue with medication? (yes/no)
Example: Memantine	Dementia medication	Daily	01.02.2019	Headaches	Yes

## APPENDIX B IPDASi v4.0 checklist 32

**Table:**

Item	Included
*Qualifying criteria*	
Describes health condition or problem	X
Explicitly states the decision that needs to be considered	X
Describes the options available	X
Describes the positive features (benefits/advantages) of each option	X
Describes the negative features (harms, side effects or disadvantages) of each option	X
Describes what it is like to experience the consequences of the options (physical, psychological, social)	X
*Certification criteria*	
Shows the negative and positive features of options in equal detail	X
Provides citations to the evidence selected	X
Provides a publication date	X
Provides an update policy	
Provides information about the levels of uncertainty around event or outcome probabilities	
Provides information about the funding source used for development	X
Describes what the test is designed to measure	N/A
Describes the next steps typically taken if the test detects the condition	N/A
Describes the next steps if the condition is not detected	N/A
Has information about the consequences of detecting the condition that would never have occurred if screening had not been done (lead time bias)	N/A
*Quality criteria*	
The patient decision aid describes the natural course of the health condition or problem, if no action is taken	X
The patient decision aid makes it possible to compare the positive and negative features of the available options	X
The patient decision aid provides information about outcome probabilities associated with the options (i.e., the likely consequences of decisions).	X
The patient decision aid specifies the defined group (reference class) of patients for whom the outcome probabilities apply.	
The patient decision aid specifies the event rates for the outcome probabilities	
The patient decision aid allows the user to compare outcome probabilities across options using the same time period (when feasible).	
The patient decision aid allows the user to compare outcome probabilities across options using the same denominator (when feasible).	
The patient decision aid provides more than 1 way of viewing the probabilities (e.g., words, numbers and diagrams).	X
The patient decision aid asks patients to think about which positive and negative features of the options matter most to them (implicitly or explicitly).	X
The patient decision aid provides a step‐by step way to make a decision.	X
The patient decision aid includes tools like worksheets or lists of questions to use when discussing options with a practitioner.	X
The development process included a needs assessment with clients or patients.	X
The development process included a needs assessment with health professionals.	X
The development process included review by clients/patients not involved in producing the decision support intervention.	X
The development process included review by professionals not involved in producing the decision support intervention.	X
The patient decision aid was field tested with patients who were facing the decision.	
The patient decision aid was field tested with practitioners who counsel patients who face the decision.	
The patient decision aid (or associated documentation) describes how research evidence was selected or synthesized.	X
The patient decision aid (or associated documentation) describes the quality of the research evidence used.	
The patient decision aid includes authors'/developers' credentials or qualifications.	X
The patient decision aid (or associated documentation) reports readability levels.	
There is evidence that the patient decision aid improves the match between the preferences of the informed patient and the option that is chosen.	N/A
There is evidence that the patient decision aid helps patients improve their knowledge about options' features.	N/A
The patient decision aid includes information about the chances of having a true‐positive test result.	N/A
The patient decision aid includes information about the chances of having a true‐negative test result.	N/A
The patient decision aid includes information about the chances of having a false‐positive test result.	N/A
The patient decision aid includes information about the chances of having a false‐negative test result.	N/A
The patient decision aid describes the chances that the disease is detected with and without the use of the test.	N/A

## References

[1] Prince M, Prina M, Guerchet M. World Alzheimer Report 2013. Journey of caring: An analysis of long‐term care for dementia. London; 2013.

[2] Alzheimer's Society. *Dementia UK: Second edition*. London; 2014.

[3] Lewis F, Karlsberg Schaffer S, Sussex J, O'Neill P, Cockcroft L. *The Trajectory of Dementia in the UK ‐ Making a Differ*ence. London; 2014.

[4] Davies N, Rait G, Maio L, Iliffe S. Family caregivers' conceptualisation of quality end‐of‐life care for people with dementia: a qualitative study. Palliat Med. 2016;31:726‐733. 27815555 10.1177/0269216316673552 PMC5625846

[5] Molyneaux V, Butchard S, Simpson J, Murray C. Reconsidering the term ‘carer': a critique of the universal adoption of the term ‘carer'. Ageing Soc. 2011;31(03):422‐437.

[6] Daly RL, Bunn F, Goodman C. Shared decision‐making for people living with dementia in extended care settings: a systematic review. BMJ Open. 2018;8(6):018977. 10.1136/bmjopen-2017-018977 PMC6009462 29886439

[7] Wendrich‐van Dael A, Bunn F, Lynch J, Pivodic L, Van den Block L, Goodman C. Advance care planning for people living with dementia: an umbrella review of effectiveness and experiences. Int J Nurs Stud. 2020;107:103576. 32380259 10.1016/j.ijnurstu.2020.103576

[8] Dixon J, Karagiannidou M, Knapp M. The effectiveness of advance care planning in improving end‐of‐life outcomes for people with dementia and their carers: a systematic review and critical discussion. J Pain Symptom Manage. 2018;55(1):132‐150. 28827062 10.1016/j.jpainsymman.2017.04.009

[9] Department of Health . Mental Capacity Act. London, UK: Stationary Office; 2005.

[10] Davies N, Rait G, Maio L, Iliffe S. Family caregivers' conceptualisation of quality end‐of‐life care for people with dementia: a qualitative study. Palliat Med. 2017;31(8):726‐733. 27815555 10.1177/0269216316673552 PMC5625846

[11] Davies N, Maio L, Rait G, Iliffe S. Quality end‐of‐life care for dementia: what have family carers told us so far? A narrative synthesis. Palliat Med. 2014;28(7):919‐930. 24625567 10.1177/0269216314526766 PMC4232347

[12] Livingston G, Leavey G, Manela M, et al. Making decisions for people with dementia who lack capacity: qualitative study of family carers in UK. BMJ. 2010;341:341. 10.1136/bmj.c4184 PMC2923693 20719843

[13] Gessert CE, Forbes S, Bern‐Klug M. Planning end‐of‐life care for patients with dementia: roles of families and health professionals. OMEGA‐J Death Dying. 2001;42(4):273‐291. 10.2190/2mt2-5gyu-gxvv-95ne 12569923

[14] Forbes S, Bern‐Klug M, Gessert C. End‐of‐life decision making for nursing home residents with dementia. Image J Nurs Scholarsh. 2000;32(3):251‐258. 10.1111/j.1547-5069.2000.00251.x 12462819

[15] Raymond, Warner A, Davies N, Manthorpe J, Ahmedzhai S, Iliffe S. Palliative care services for people with dementia: a synthesis of the literature reporting the views and experiences of professionals and family carers. Dementia: Int J Soc Stud. 2014;13:96‐110. 10.1177/1471301212450538 24381041

[16] Davies N, Schiowitz B, Rait G, Vickerstaff V, Sampson EL. Decision aids to support decision making in dementia care: a systematic review. Int Psychogeriaatrics. 2019;31:1403‐1419. 10.1017/S1041610219000826 31347482

[17] Lord K, Livingston G, Cooper C. A systematic review of barriers and facilitators to and interventions for proxy decision‐making by family carers of people with dementia. Int Psychogeriatr. 2015;27(08):1301‐1312. 25870004 10.1017/S1041610215000411

[18] Brazil K, Carter G, Cardwell C, et al. Effectiveness of advance care planning with family carers in dementia nursing homes: a paired cluster randomized controlled trial. Palliat Med. 2018;32(3):603‐612. 28786323 10.1177/0269216317722413

[19] Stacey D, Légaré F, Lewis K, et al. Decision aids for people facing health treatment or screening decisions. Cochrane Libr. 2017;4. 10.1002/14651858.CD001431.pub5 PMC6478132 28402085

[20] Stacey D, Légaré F, Boland L, et al. 20th Anniversary Ottawa Decision Support Framework: Part 3 overview of systematic reviews and updated framework. Med Decis Making. 2020;40(3):379‐398. 32428429 10.1177/0272989X20911870

[21] Davies N, Iliffe S. End of life care—why those with dementia have different needs. BMJ. 2016;353(i2171):2171. 10.1136/bmj.i2171 27090749

[22] Geddis‐Regan A, Errington L, Abley C, Wassall R, Exley C, Thomson R. Enhancing shared and surrogate decision making for people living with dementia: a systematic review of the effectiveness of interventions. Health Expect. 2020;24(1):19‐32. 33248009 10.1111/hex.13167 PMC7879553

[23] Hanson LC, Carey TS, Caprio AJ, et al. Improving decision‐making for feeding options in advanced dementia: a randomized, controlled trial. J Am Geriatr Soc. 2011;59(11):2009‐2016. 22091750 10.1111/j.1532-5415.2011.03629.x PMC3227016

[24] Lord K, Livingston G, Cooper C. A feasibility randomised controlled trial of the DECIDE intervention: dementia carers making informed decisions. BJPsych Open. 2017;3(1):12‐14. 28243460 10.1192/bjpo.bp.116.003509 PMC5288639

[25] Stirling C, Leggett S, Lloyd B, et al. Decision aids for respite service choices by carers of people with dementia: development and pilot RCT. BMC Med Inform Decis Mak. 2012;12(1):1‐10. 22429384 10.1186/1472-6947-12-21 PMC3315425

[26] Hanson LC, Zimmerman S, Song M‐K, et al. Effect of the goals of care intervention for advanced dementia: a randomized clinical trial. JAMA Intern Med. 2017;177(1):24‐31. 27893884 10.1001/jamainternmed.2016.7031 PMC5234328

[27] Volandes AE, Paasche‐Orlow MK, Barry MJ, et al. Video decision support tool for advance care planning in dementia: randomised controlled trial. BMJ. 2009;338:338. 10.1136/bmj.b2159 PMC2688013 19477893

[28] Coulter A, Stilwell D, Kryworuchko J, Mullen PD, Ng CJ, van der Weijden T. A systematic development process for patient decision aids. BMC Med Inform Decis Mak. 2013;13(2):1‐7. 24625093 10.1186/1472-6947-13-S2-S2 PMC4044159

[29] Davies N, De Souza T, Rait G, Meehan J, Sampson EL. Developing an applied model for making decisions towards the end of life about care for someone with dementia. PLOS One. 2021;16(5):e0252464. 34043728 10.1371/journal.pone.0252464 PMC8158904

[30] Stacey D, Ludwig C, Archambault P, et al. Feasibility of rapidly developing and widely disseminating patient decision aids to respond to urgent decisional needs due to the COVID‐19 pandemic. Med Decis Making. 2020;41(2):233‐239. 33300438 10.1177/0272989X20979693 PMC7879222

[31] Rapaport P, Webster L, Horsley R, et al. An intervention to improve sleep for people living with dementia: reflections on the development and co‐production of DREAMS:START (Dementia RElAted Manual for Sleep: sTrAtegies for RelaTives). Dementia. 2018;17(8):976‐989. 30373463 10.1177/1471301218789559

[32] Davies N, Hopwood J, Walker N, et al. Designing and developing a co‐produced theoretical and evidence‐based online support for family caregivers of people with dementia at the end of life. BMC Palliat Care. 2019;18(1):71. 31409329 10.1186/s12904-019-0455-0 PMC6693100

[33] Davies N, Mathew R, Wilcock J, et al. A co‐design process developing heuristics for practitioners providing end of life care for people with dementia. BMC Palliat Care. 2016;15(68):68. 27484683 10.1186/s12904-016-0146-z PMC4969644

[34] O'Cathain A, Croot L, Sworn K, et al. Taxonomy of approaches to developing interventions to improve health: a systematic methods overview. Pilot Feasibility Stud. 2019;5(1):41. 30923626 10.1186/s40814-019-0425-6 PMC6419435

[35] Elwyn G, Kreuwel I, Durand MA, et al. How to develop web‐based decision support interventions for patients: a process map. Patient Educ Couns. 2011;82(2):260‐265. 20627644 10.1016/j.pec.2010.04.034

[36] O'Connor AM, Tugwell P, Wells GA, et al. A decision aid for women considering hormone therapy after menopause: decision support framework and evaluation. Patient Educ Couns. 1998;33(3):267‐279. 9731164 10.1016/s0738-3991(98)00026-3

[37] Légaré F, Stacey D, Gagnon S, et al. Validating a conceptual model for an inter‐professional approach to shared decision making: a mixed methods study. J Eval Clin Pract. 2011;17(4):554‐564. 20695950 10.1111/j.1365-2753.2010.01515.x PMC3170704

[38] The Patient Decision Aids Research Group. *Interprofessional Shared Decision Making (IP‐SDM) Model*; 2021. https://decisionaid.ohri.ca/ip-sdm.html. Accessed February 2, 2021.

[39] Elwyn G, O'connor A, Stacey D, et al. Developing a quality criteria framework for patient decision aids: online international Delphi consensus process. BMJ. 2006;333(7565):417. 16908462 10.1136/bmj.38926.629329.AE PMC1553508

[40] Waite J, Poland F, Charlesworth G. Facilitators and barriers to co‐research by people with dementia and academic researchers: findings from a qualitative study. Health Expect. 2019;22(4):761‐771. 31012214 10.1111/hex.12891 PMC6737841

[41] Bethell J, Commisso E, Rostad HM, et al. Patient engagement in research related to dementia: s scoping review. Dementia. 2018;17(8):944‐975. 30373460 10.1177/1471301218789292

[42] Van de Ven AH, Delbecq AL. The nominal group as a research instrument for exploratory health studies. Am J Public Health. 1972;62(3):337‐342. 5011164 10.2105/ajph.62.3.337 PMC1530096

[43] Davies N, Manthorpe J, Sampson EL, Iliffe S. After the Liverpool Care Pathway—development of heuristics to guide end of life care for people with dementia: protocol of the ALCP study. BMJ Open. 2015;5(9):008832. 10.1136/bmjopen-2015-008832 PMC4563245 26338688

[44] Van Someren M, Barnard Y, Sandberg J. The Think Aloud Method: A Practical Approach to Modelling Cognitive. London: Academic Press; 1994.

[45] McCarron TL, Noseworthy T, Moffat K, et al. A co‐designed framework to support and sustain patient and family engagement in health‐care decision making. Health Expect. 2020;23:825‐836. 32337836 10.1111/hex.13054 PMC7495064

[46] Grindell C, Tod A, Bec R, et al. Using creative co‐design to develop a decision support tool for people with malignant pleural effusion. BMC Med Inform Decis Mak. 2020;20(1):1‐12. 32758243 10.1186/s12911-020-01200-3 PMC7404910

[47] Shepherd V, Wood F, Griffith R, Sheehan M, Hood K. Development of a decision support intervention for family members of adults who lack capacity to consent to trials. BMC Med Inform Decis Mak. 2021;21(1):30. 33509169 10.1186/s12911-021-01390-4 PMC7842028

[48] Carter G, McLaughlin D, Kernohan WG, et al. The experiences and preparedness of family carers for best interest decision‐making of a relative living with advanced dementia: a qualitative study. J Adv Nurs. 2018;74(7):1595‐1604. 29603347 10.1111/jan.13576

[49] van der Steen JT, Radbruch L, Hertogh CM, et al. White paper defining optimal palliative care in older people with dementia: a Delphi study and recommendations from the European Association for Palliative Care. Palliat Med. 2014;28(3):197‐209. 23828874 10.1177/0269216313493685

[50] Jones K, Birchley G, Huxtable R, Clare L, Walter T, Dixon J. End of life care: a scoping review of experiences of advance care planning for people with dementia. Dementia. 2019;18(3):825‐845. 27821714 10.1177/1471301216676121

[51] Tuijt R, Rees J, Frost R, et al. Exploring how triads of people living with dementia, carers and health care professionals function in dementia health care: a systematic qualitative review and thematic synthesis. Dementia. 2020;20(3):1080‐1104. 32212862 10.1177/1471301220915068 PMC8047709

[52] Moore K, Sampson E, Kupeli N, Davies N. Supporting families in end‐of‐life care and bereavement in the COVID‐19 era. Int Psychogeriatr. 2020;32:1‐10. 32349850 10.1017/S1041610220000745 PMC7235296

[53] Abhyankar P, Volk RJ, Blumenthal‐Barby J, et al. Balancing the presentation of information and options in patient decision aids: an updated review. BMC Med Inform Decis Mak. 2013;13(Suppl 2):S6. 10.1186/1472-6947-13-S2-S6 PMC4044010 24625214

[54] Pickett J, Murray M. Editorial: Patient and public involvement in dementia research: setting new standards. Dementia. 2018;17(8):939‐943. 30373464 10.1177/1471301218789290

[55] Craig P, Dieppe P, Macintyre S, Michie S, Nazareth I, Petticrew M. Developing and evaluating complex interventions: the new Medical Research Council guidance. BMJ. 2008;337:a1655. 18824488 10.1136/bmj.a1655 PMC2769032

[56] Joseph‐Williams N, Abhyankar P, Boland L, et al. What works in implementing patient decision aids in routine clinical settings? A rapid realist review and update from the international patient decision aid standards collaboration. Med Decis Making. 2020;0:272989X20978208. 10.1177/0272989X20978208 PMC8474331 33319621

[57] England NHS . *The NHS Long Term Plan*. London; 2019.

